# Supplementary material for: Sequential production of gametes during meiosis in trypanosomes
Source: Commun Biol. 2021 May 11;4:555. doi: 10.1038/s42003-021-02058-5 (PMC8113336; doi:10.1038/s42003-021-02058-5)
Supplement: Supplementary file 2 — Supplementary Information [file 42003_2021_2058_MOESM2_ESM.docx]

**Supplementary Information**

**Supplementary Figure 1** Copy number of *H2B::GFP* gene in *T. b. brucei* 1738

QPCR was used to determine the copy number of *GFP* and *YFP* relative to the endogenous single-copy gene encoding triose phosphate isomerase (*TIM*) [1] in *T. b. brucei* 1738 using QPCR as described previously [2]; both *GFP* and *YFP* genes were amplified with the same primer set. All QPCR reactions were performed in triplicate with positive (*T. b. brucei* 1738 *GFP*) and negative (*T. b. brucei* 1738 wildtype) DNA controls. DNA of transfected clones 1738 *H2B::GFP*  and 1738 *H2B::GFP*  *PFR1::YFP* were tested and resulting data were analysed using MX Pro software (Agilent Technologies). The ratio for 1738 *H2B::GFP*  was approximately 1:2 *GFP:TIM* and for 1738 *H2B::GFP*  *PFR1::YFP* it was approximately 1:1 (Figure 1), indicating that there is a single copy of *H2B::GFP* in 1738 *H2B::GFP*, and single copies of both *H2B::GFP* and *PFR::YFP* in 1738 *H2B::GFP*  *PFR1::YFP*.


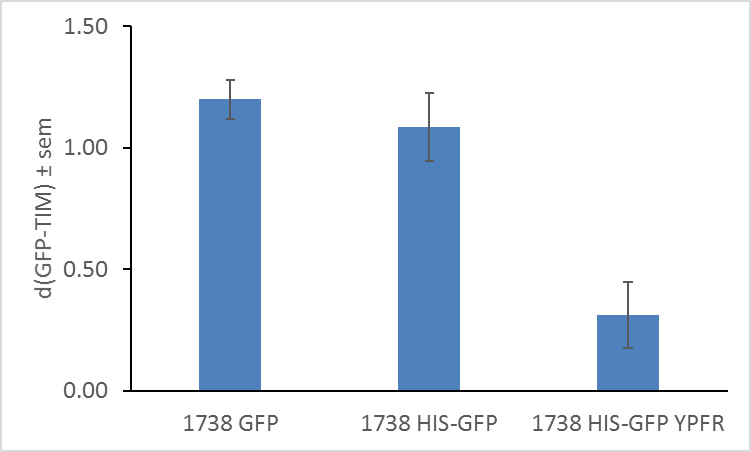


-----------------------

**Supplementary Table 1** Categorisation of all trypanosome cell types in the salivary gland-derived population.

Combined results for *T. b. brucei* J10 *YFP::HOP1 PFR1::YFP* and J10 *YFP::DMC1 PFR1::YFP*. Meiotic dividers were identified by expression of *YFP::DMC1* or *YFP::HOP1*.

| **Morphology** | **No.** | **%** |
| --- | --- | --- |
| Trypomastigote | 11 | 5 |
| Dividing trypomastigote | 6 | 3 |
| Epimastigote | 29 | 12 |
| Dividing epimastigote | 19 | 8 |
| Epimastigote expressing DMC/HOP = Meiotic divider | 17 | 7 |
| Metacyclic | 13 | 6 |
| Gamete | 72 | 31 |
| Potential meiotic intermediates | 50 | 21 |
| Uncategorised | 19 | 8 |
| Total | 236 | 100 |

**Supplementary Table 2** Characteristics of 3N and 2N trypanosomes with 1C nuclei identified in SG-derived populations of 1738 *H2B::GFP* *PFR1::YFP*.

| No. of nuclei | 2C nuclei | 1C nuclei | Description | No. | Total |
| --- | --- | --- | --- | --- | --- |
| 3 | 1 | 2 | Order of nuclei from cell posterior: 1C, 1C, 2C | 8 | 39 |
|  |  |  | Order of nuclei from cell posterior: 1C, 2C, 1C | 19 |  |
|  |  |  | Order of nuclei from cell posterior: 2C, 1C, 1C | 3 |  |
|  |  |  | Order of nuclei from cell posterior: nuclei adjacent | 9 |  |
|  |  |  | Cytokinesis: 1K1N gamete with 1C nucleus | 7 | 13 |
|  |  |  | Cytokinesis: 2K1N gamete with 1C nucleus | 6 |  |
|  |  |  |  |  |  |
| 2 | 1 | 1 | Order of nuclei from cell posterior: 1C, 2C | 43 | 87 |
|  |  |  | Order of nuclei from cell posterior: 2C, 1C | 34 |  |
|  |  |  | Order of nuclei from cell posterior: nuclei adjacent | 10 |  |
|  |  |  | Cytokinesis: new 2K1N gamete with 1C nucleus | 3 | 3 |

**Supplementary Table 3** Morphology of potential meiotic intermediates among SG-derived trypanosomes of 1738 *H2B::GFP* *PFR1::YFP*. Designation of intermediates as in **Figure 7** model of meiosis.

| **No. of nuclei** | **1K** | **2K** | **3K** | **4K** | **5K** | **6K** | **Total** |
| --- | --- | --- | --- | --- | --- | --- | --- |
| 1N (intermediates E1 and E2) |  | 13 | 17 |  |  |  | 30 |
| 2N (2x 2C; intermediate B) |  | 8 | 20 | 29 | 1 | 1 | 59 |
| 2N (2x 1C; intermediates F1 and F2) | 4 | 14 | 18 |  |  |  | 36 |
| 2N (1x 2C, 1x 1C; intermediates D1 and D2) | 20 | 48 | 15 | 4 |  |  | 87 |
| 3N (1x 2C, 2x 1C; intermediates C1 and C2) | 3 | 9 | 19 | 16 | 3 | 2 | 52 |
| Total | 27 | 92 | 89 | 49 | 4 | 3 | 264 |

**Supplementary references**

1. Gibson, W.C., Osinga, K.A., Michels, P.A.M., and Borst, P. (1985). Trypanosomes of subgenus *Trypanozoon* are diploid for housekeeping genes. Mol. Biochem. Parasitol. *16*, 231-242.

2. Gibson, W., Peacock, L., Ferris, V., Fischer, K., Livingstone, J., Thomas, J., and Bailey, M. (2015). Genetic recombination between human and animal parasites creates novel strains of human pathogen. PLoS NTD *9*, e0003665.
