# Supplementary material for: Sequential production of gametes during meiosis in trypanosomes
Source: Commun Biol. 2021 May 11;4:555. doi: 10.1038/s42003-021-02058-5 (PMC8113336; doi:10.1038/s42003-021-02058-5)
Supplement: Supplementary file 3 — Description of Supplementary Files [file 42003_2021_2058_MOESM3_ESM.pdf]

Description of Additional Supplementary Files

File name: Supplementary Data 1

Description: Excel file containing mensural data for individual cells.
