# Supplementary material for: Sequential production of gametes during meiosis in trypanosomes
Source: Commun Biol. 2021 May 11;4:555. doi: 10.1038/s42003-021-02058-5 (PMC8113336; doi:10.1038/s42003-021-02058-5)
Supplement: Supplementary file 5 — Reporting Summary [file 42003_2021_2058_MOESM5_ESM.pdf]

nature research

Corresponding author(s): DBPR COMMSBIO-20-1291-T

Last updated by author(s): Mar 19, 2021

Reporting Summary

Nature Research wishes to improve the reproducibility of the work that we publish. This form provides structure for consistency and transparency in reporting. For further information on Nature Research policies, see our Editorial Policies and the Editorial Policy Checklist.

Statistics

For all statistical analyses, confirm that the following items are present in the figure legend, table legend, main text, or Methods section.

n/a	Confirmed	
☐	☒	The exact sample size (*n*) for each experimental group/condition, given as a discrete number and unit of measurement
☐	☒	A statement on whether measurements were taken from distinct samples or whether the same sample was measured repeatedly
☐	☒	The statistical test(s) used AND whether they are one- or two-sided<br>*Only common tests should be described solely by name; describe more complex techniques in the Methods section.*
☒	☐	A description of all covariates tested
☒	☐	A description of any assumptions or corrections, such as tests of normality and adjustment for multiple comparisons
☐	☒	A full description of the statistical parameters including central tendency (e.g. means) or other basic estimates (e.g. regression coefficient) AND variation (e.g. standard deviation) or associated estimates of uncertainty (e.g. confidence intervals)
☒	☐	For null hypothesis testing, the test statistic (e.g. *F*, *t*, *r*) with confidence intervals, effect sizes, degrees of freedom and *P* value noted<br>*Give P values as exact values whenever suitable.*
☒	☐	For Bayesian analysis, information on the choice of priors and Markov chain Monte Carlo settings
☒	☐	For hierarchical and complex designs, identification of the appropriate level for tests and full reporting of outcomes
☒	☐	Estimates of effect sizes (e.g. Cohen's *d*, Pearson's *r*), indicating how they were calculated

Our web collection on statistics for biologists contains articles on many of the points above.

Software and code

Policy information about availability of computer code

Data collection	Digital microscope images acquired using Volocity software (PerkinElmer).
Data analysis	Details of all software used for data analysis is included in Methods section of main paper or supplementary material. ImageJ (http://rsb.info.nih.gov/ij) was used for image analysis. Excel (Microsoft) was used to prepare graphs. MX Pro software (Agilent Technologies) was used to analyse QPCR data.

For manuscripts utilizing custom algorithms or software that are central to the research but not yet described in published literature, software must be made available to editors and reviewers. We strongly encourage code deposition in a community repository (e.g. GitHub). See the Nature Research guidelines for submitting code & software for further information.

Data

Policy information about availability of data

All manuscripts must include a data availability statement. This statement should provide the following information, where applicable:

- Accession codes, unique identifiers, or web links for publicly available datasets
- A list of figures that have associated raw data
- A description of any restrictions on data availability

All data are contained in this publication and supplementary files.

Field-specific reporting

Please select the one below that is the best fit for your research. If you are not sure, read the appropriate sections before making your selection.

- [x] Life sciences
- [ ] Behavioural & social sciences
- [ ] Ecological, evolutionary & environmental sciences

For a reference copy of the document with all sections, see nature.com/documents/nr-reporting-summary-flat.pdf

Life sciences study design

All studies must disclose on these points even when the disclosure is negative.

Sample size	No sample size calculation performed. Experimental tsetse transmissions were repeated as necessary to obtain large numbers of cells for analysis and generate meaningful results, generally >100 cells.
Data exclusions	Trypanosomes with cell morphologies that could not be categorized were excluded from the analysis; it is stated in the text and/or table legends where this was done.
Replication	Experiments were repeated with different trypanosome strains and with different recombinant clones. In addition, each set of image data derives from several replicate tsetse transmission experiments.
Randomization	Tsetse transmission experiments were carried out batch-wise, depending on the availability of newly emerged flies. Conditions for infection, maintenance and dissection were kept as consistent as possible.
Blinding	Blinding was not relevant to the study as there were not negative control groups of infected flies or different treatment groups.

Reporting for specific materials, systems and methods

We require information from authors about some types of materials, experimental systems and methods used in many studies. Here, indicate whether each material, system or method listed is relevant to your study. If you are not sure if a list item applies to your research, read the appropriate section before selecting a response.

Materials & experimental systems

n/a	Involved in the study	
x		Antibodies
	x	Eukaryotic cell lines
x		Palaeontology and archaeology
	x	Animals and other organisms
x		Human research participants
x		Clinical data
x		Dual use research of concern

Methods

n/a	Involved in the study	
x		ChIP-seq
x		Flow cytometry
x		MRI-based neuroimaging

Eukaryotic cell lines

Policy information about cell lines

Cell line source(s)	Trypanosome cell lines (1738, J10) were sourced from the Bristol Trypanosome Research group (Wendy Gibson, School of Biological Sciences, University of Bristol, UK). Other genetically modified trypanosome clones (TREU 927, STIB 247) were obtained from Eva Gluenz and Keith Gull (Sir William Dunn School of Pathology, Oxford University, Oxford, UK).
Authentication	Recombinant trypanosome lines were cloned and their ability to complete the tsetse transmission cycle was confirmed before experiments were done.
Mycoplasma contamination	Not tested. Commercial FCS used in media preparation was negative for Mycoplasma contamination.
Commonly misidentified lines (See ICLAC register)	No commonly misidentified cell lines were used in this work.

Animals and other organisms

Policy information about studies involving animals; ARRIVE guidelines recommended for reporting animal research

Laboratory animals	Tsetse flies (Glossina pallidipes) male and female adult flies. Sourced as pupae from laboratory colonies maintained at IAEA, Vienna, Austria.

April 2020

Wild animals	No wild animals used in this study.
Field-collected samples	Study did not involve animals collected from the field.
Ethics oversight	No ethical permission is required for tsetse experiments as they are insects.

Note that full information on the approval of the study protocol must also be provided in the manuscript.
